# The Role of Porcine Monocyte Derived Dendritic Cells (MoDC) in the Inflammation Storm Caused by *Streptococcus suis* Serotype 2 Infection

**DOI:** 10.1371/journal.pone.0151256

**Published:** 2016-03-14

**Authors:** Jin Liu, Zhong-Yuan Tian, Yun-Cai Xiao, Xi-Liang Wang, Mei-Lin Jin, De-Shi Shi

**Affiliations:** 1 State Key Laboratory of Agricultural Microbiology, College of Veterinary Medicine, Huazhong Agricultural University, Wuhan, 430070, Hubei, China; 2 Key Laboratory of Development of Veterinary Diagnostic Products of Ministry of Agricultural, College of Veterinary Medicine, Huazhong Agricultural University, Wuhan, 430070, Hubei, China; University of Leuven, Rega Institute, BELGIUM

## Abstract

**Background:**

*Streptococcus suis* is an important swine pathogen and zoonotic agent. Infection with this highly pathogenic strain can cause streptococcal toxic shock-like syndrome (STSLS), characterized by a Th-1 inflammatory cytokine storm, and a high mortality rate. Monocyte derived dendritic cells (MoDCs) are known to stimulate Th-1 cell differentiation, but the role of MoDCs in STSLS remains to be elucidated.

**Methodology and Findings:**

Porcine CD14-positive monocytes, purified from peripheral blood mononuclear cells (PBMCs), were used to generate MoDCs using granulocyte-macrophage colony-stimulating factor (GM-CSF) and interleukin-4 (IL-4). Highly pure MoDCs were generated, as proved by their morphology, phenotype analysis, phagocytic ability, and induction of T cells proliferation. The MoDCs were further stimulated by the virulent S. suis serotype 2 (SS2) SC19 strain which triggered a strong release of several pro-inflammatory cytokines, including IL-1β, IL-8, TNF-α, IFN-γ, and IL-12. Furthermore, the stimulated MoDCs induced CD4^+^ T cell differentiation towards Th-1 cells *in vitro*.

**Conclusions:**

The results of this study indicated that the porcine MoDCs stimulated by SS2 could release high levels of Th-1 inflammatory cytokines and induce CD4^+^ T cell differentiation towards Th-1 cells. Hence, it is likely that porcine MoDCs play an important role in the STSLS caused by SS2.

## Introduction

*Streptococcus suis* (*S*.*suis*) is a major swine pathogen, causing considerable economic losses and animal health care problems in the pig farming industry worldwide [1]. *S*.*suis* infection causes several diseases, including meningitis and septicemia—the main causes of mortality with this infection. *S*.*suis* is also an emerging serious zoonotic pathogen of humans, particularly in Asia [2, 3].

The capsule is the most important virulence factor in *S*.*suis* [4]. Out of the 33 serotypes of *S*.*suis*, serotype 2 (SS2) is the most common and most virulent serotype. It is associated with diseases in humans and pigs in most of the countries [5, 6]. In 2005, a large outbreak of human SS2 infection occurred in Sichuan, China, resulting in 215 infections and 38 deaths [7]. During this outbreak, some people presented with streptococcal toxic shock-like syndrome (STSLS) and more than 80% of the patients died in spite of treatment with antibiotics [8,9]. Furthermore, the clinical surveys indicated that the patients who died had high levels of the Th-1 inflammatory cytokines in their serum. Subsequent studies have confirmed that extremely high levels of the Th-1 inflammatory cytokines are an important characteristic of STSLS [10].

Monocyte derived dendritic cells (MoDCs) can stimulate Th-1 cell differentiation. Dendritic cells (DCs) can be activated by invading microbes. Activated DCs produce various cytokines that can regulate inflammation and direct the differentiation of the CD4^+^ T cells [11, 12, 13, 14]. However, the role of MoDCs in the Th-1 response to SS2 infection is still unknown. Therefore, the present study aimed to examine the role of MoDCs in the Th-1 response to SS2 infection.

## Results

### Identification of porcine MoDCs

**Fig 1:**
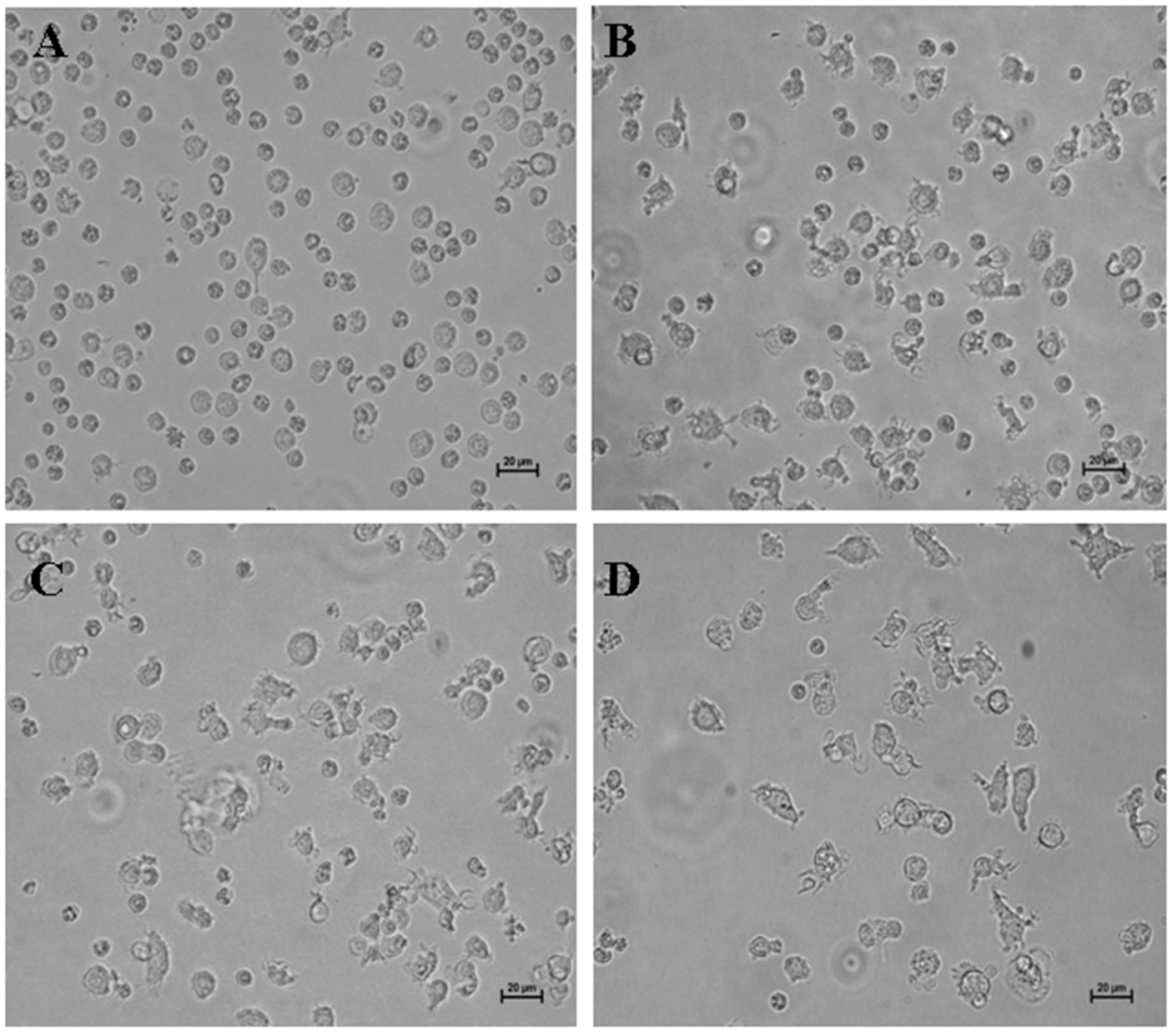
Porcine MoDCs at different culture times (400×). CD14^+^ monocytes were purified from porcine PBMCs and were cultured in RPMI-1640 medium supplemented with 10% fetal bovine serum (FBS), 25 ng/mL recombinant porcine IL-4, and 10 ng/ml recombinant porcine GM-CSF. The culture medium was replenished every 3 days. (A) CD14^+^ monocytes after 2 days of culture. (B) CD14^+^ monocytes 3 days of culture. (C) CD14^+^ monocytes after 4 days of culture. (D) CD14^+^ monocytes after 5 days of culture.

The CD14^+^ monocytes purified from porcine peripheral blood mononuclear cells (PBMCs) were small and round after being directionally cultured for two days (Fig 1A). After three days, most of the cells were big and veiled (Fig 1B). At the fourth and fifth day of culture, the cells were semi-suspended, and were characterized by a veiled morphology, that is typical of DCs (Fig 1C and 1D).

**Fig 2:**
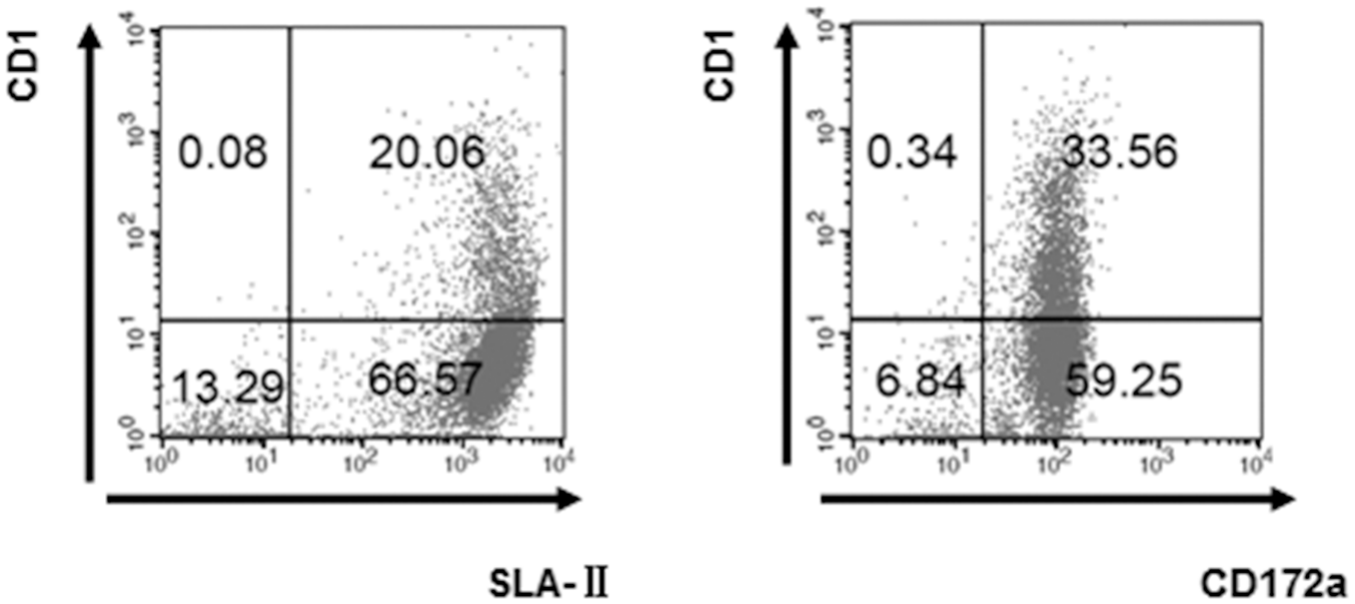
Identification of 5-day old MoDCs by FCM. 10^6^ of 5-day old MoDC cells were harvested and stained with mouse anti pig CD1, mouse anti pig SLA-II, mouse anti porcine CD172a and analyzed on a FACSCalibur flow cytometer (BD Biosciences, Hiedelberg, Germany) by using CellQuestPro software (BD Biosciences).

After being directionally cultured for five days, flow cytometric analysis showed that CD1 expression on the CD14^+^ monocytes was low (20.14%~33.90%) while swine leukocyte antigen II (SLA-II) and CD172a expression was high (86.63%, 92.81%) (Fig 2), as has been previously reported to be characteristic of MoDCs [15].

### Flow cytometry analysis of *S*.*suis-*infected MoDCs

**Fig 3:**
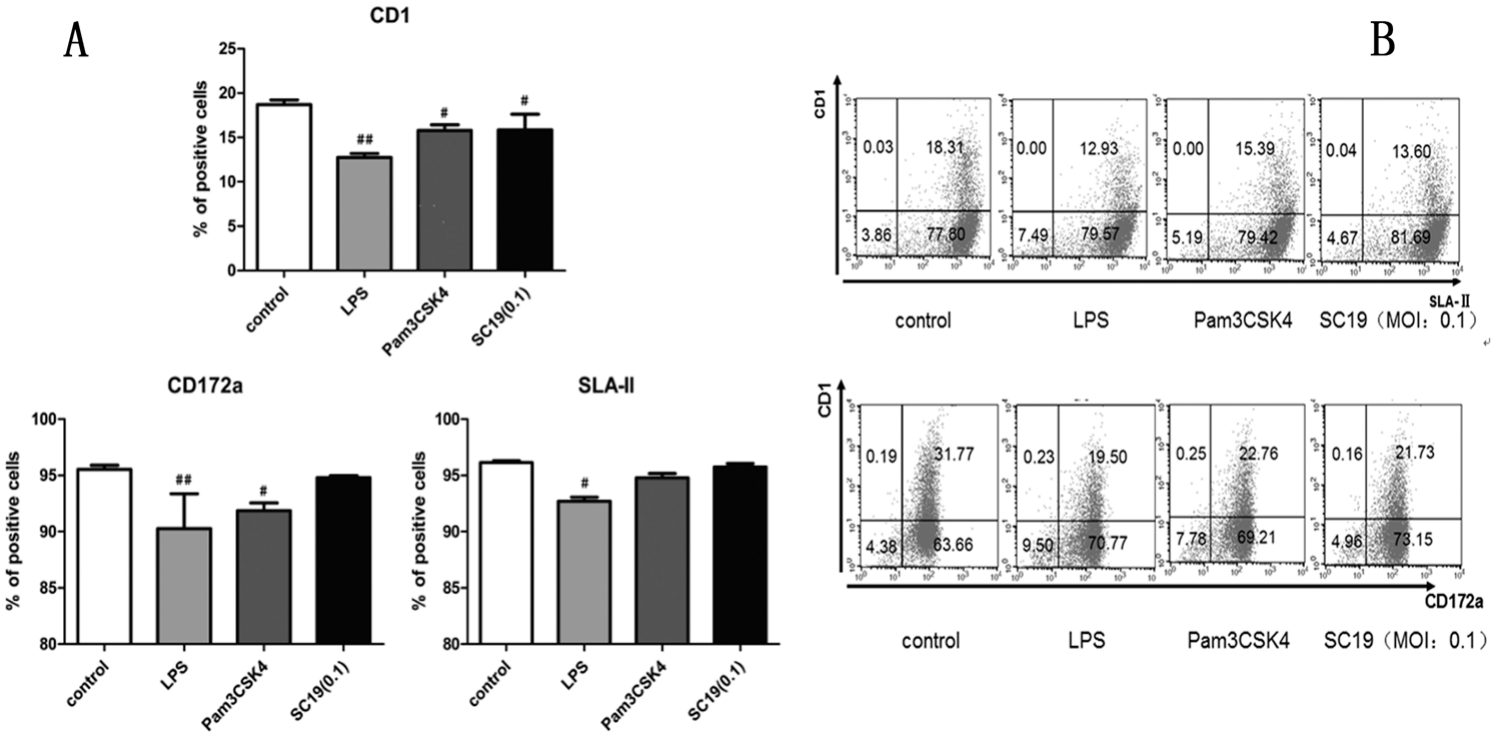
Expression levels of major surface markers on 7-day old MoDC. 6-day old MoDCs were treated with LPS (1 μg/mL), Pam3CSK4 (500 ng/mL) or SC19 (MOI = 0.1) for 24 h, and expression levels of major surface markers on MoDC were analyzed by flow cytometry. (A) Three different panels indicating the CD1, CD172a and SLA-II expression. (B) The representative dot-plot of flow cytometry analysis data for the CD1, CD172a and SLA-II expression.

The mature of MoDCs infected with *S*. *suis* was evaluated by Flow cytometry analysis. During the analysis, Pam3CSK4 and LPS, which are typical pathogen-associated molecular patterns (PAMPs) of Gram-positive or negative bacteria respectively, were used as positive controls of promoting DCs maturation. The assay indicated that 6-day old MoDCs were treated with SC19 [multiplicity of infection (MOI = 0.1] for 24 h, and their CD1 expression was found to be down-regulated by about 25%, while CD172a and SLA-II expression remained stable (Fig 3), suggesting that SC19 promoted the maturation of MoDCs.

### Phagocytic ability of MoDCs treated with neutral red

**Fig 4:**
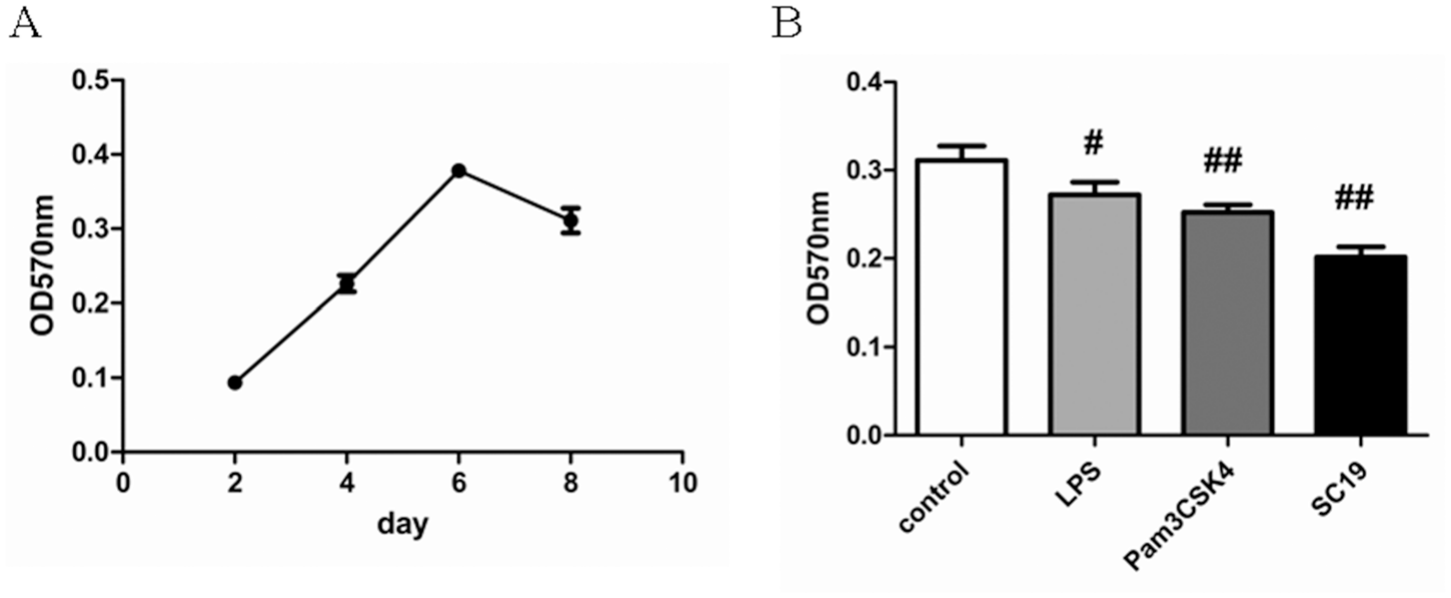
Phagocytic ability of MoDCs treated with neutral red. (A) OD570nm of MoDCs at different culture times. (B) OD570nm of 8-day old MoDCs when stimulated with SC19 (MOI = 0.1), LPS (1 μg/mL) or Pam3CSK4 (500 ng/mL) for 2 days.

To further examine whether SC19 could promote the maturation of MoDCs, the phagocytic ability of MoDCs for neutral red was tested. Phagocytic ability of the CD14^+^ monocytes at different culture times was tested, and it was found that the phagocytic ability of the cells increased until day 6, and then started to decrease (Fig 4A), which is a known characteristic of MoDCs and also indicated that most porcine MoDCs are immature before day 6. So, the 6-day old MoDCs were selected to be stimulated by SC19 for two days, the phagocytic ability of the stimulated MoDCs decreased by 35.2% (Fig 4B), suggesting that SC19 (MOI = 0.1) can promote the maturation of MoDCs.

### Proliferation of T cells stimulated by SS2-treated MoDCs

**Fig 5:**
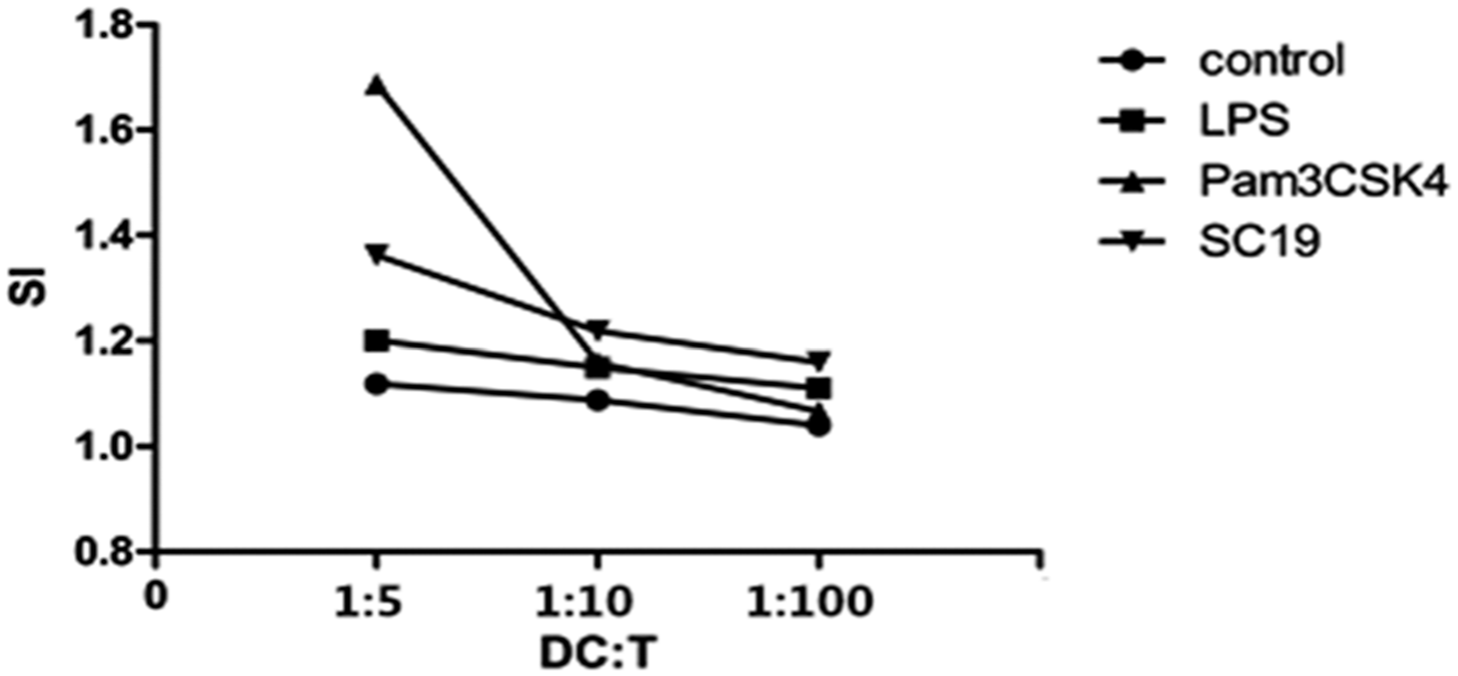
SI of T cells stimulated by MoDCs. 5-day old MoDCs were treated with SC19 (MOI = 0.001), LPS (1 μg/mL) or Pam3CSK4 (500 ng/mL) for 24 h, and then co-cultured with T cells in different ratios. Three days later, OD450nm of each well was detected by microplate reader and SI was calculated.

When T cells were co-cultured with pre-treated MoDCs for three days, the Stimulation Index (SI) of the T cells being co-cultured with SC19-stimulated MoDCs was higher than that of those being co-cultured with control and lipopolysaccharide (LPS) -stimulated MoDCs. Moreover, the SI showed a dose-response relationship, (Fig 5) suggesting that the SC19-stimulated MoDCs can enhance the proliferation of T cells.

### Cytokine production in MoDCs stimulated by SC19

**Fig 6:**
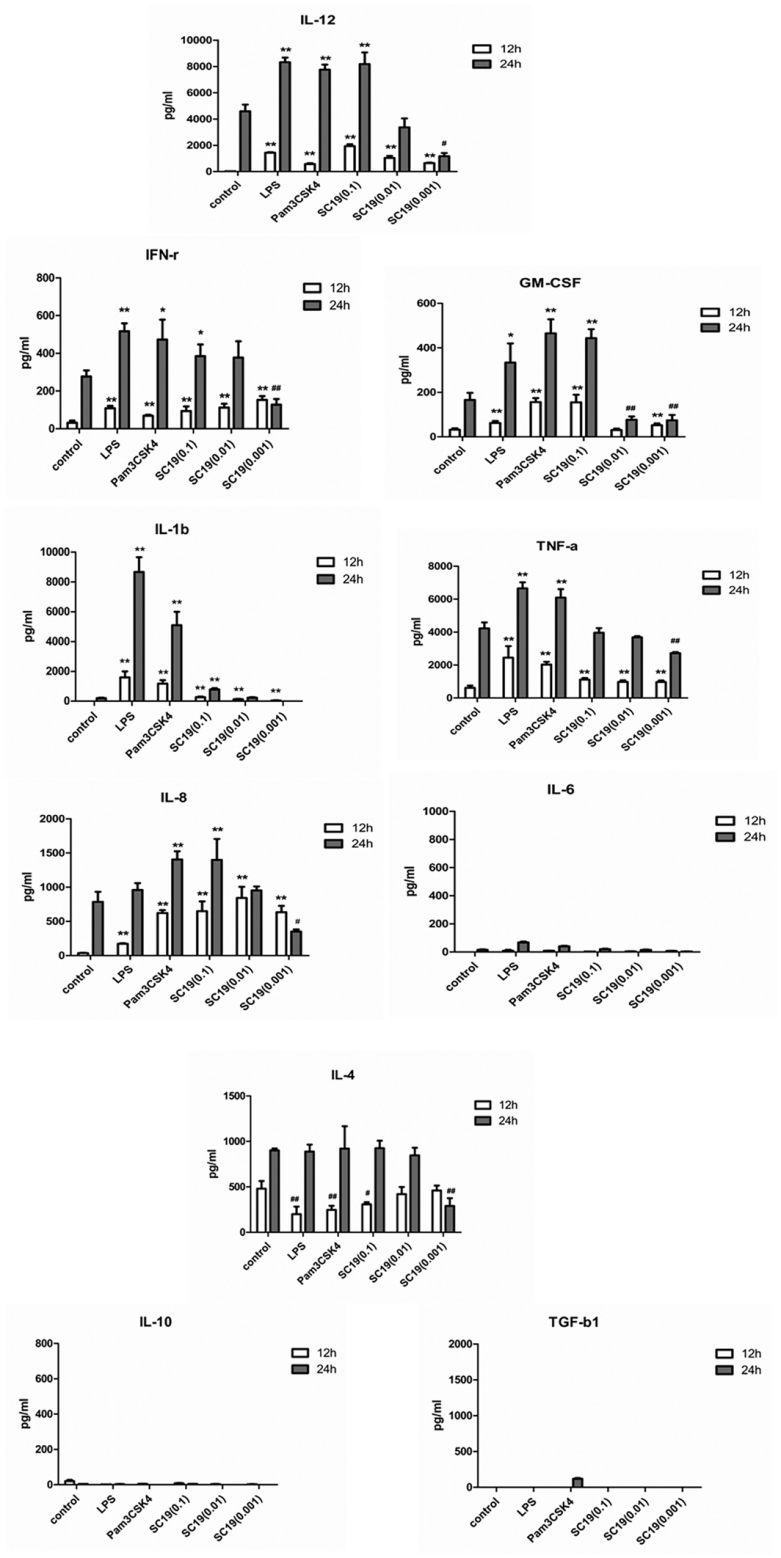
Cytokines secreted by MoDCs in response to the SC19 stimulation. 5-day old MoDCs were stimulated by SC19 (MOI: 0.1, 0.01, 0.001), LPS (1μg/mL) or Pam3CSK4 (500ng/mL). After 12 and 24 h, supernatants were collected and analyzed for cytokines using Porcine Cytokine Array.

The levels of IL-1β, IL-4, IL-6, IL-10, IL-12, tumor necrosis factor-alpha (TNF-α), Interferon-γ (IFN-γ), transforming growth factor beta (TGF-β), and GM-CSF in the supernatants of the SC19-stimulated MoDCs were measured at 12 and 24 h after stimulation and the results are shown in Fig 6. The SC19-stimulated MoDCs produced a significant amount of Th-1 inducing cytokine (IL-12) and the amount produced followed a dose-response curve. IL-12 is known to induce Th-1 cells differentiation. Thus, the results indicate that the SC19-stimulated MoDCs can induce Th-1 polarization. The levels of GM-CSF and IFN-γ were up-regulated and showed a dose-response relationship with SC19 stimulation, suggesting that the SC19 -stimulated MoDCs can promote Th-1cells amplification.

Pro-inflammatory cytokines like the IL-1β, IFN-γ, TNF-α, and IL-8 (except IL-6), released by the SC19-stimulated MoDCs were much higher (2–100 fold) than that by control and showed a dose-response relationship with SC19. It should be noted that IL-6 was not up-regulated by SC19 at all.

On the other hand, levels of TGF-β1 and IL-10 released by the SC19-stimulated MoDCs were too low to be detected. It is well known that inflammation can be suppressed by TGF-β1 and IL-10. Hence, our results suggest that the inflammation caused by SC19 was not under control. The level of IL-4 was down-regulated by SC19 in a dose dependent manner at 12 h, and remained stable at 24 h except by SC19 (MOI = 0.001), that indicated that the MoDCs stimulated by SC19 did not induce Th-2 polarization.

### CD4^+^ T cell differentiation response to SC19-stimulated MoDCs

**Fig 7:**
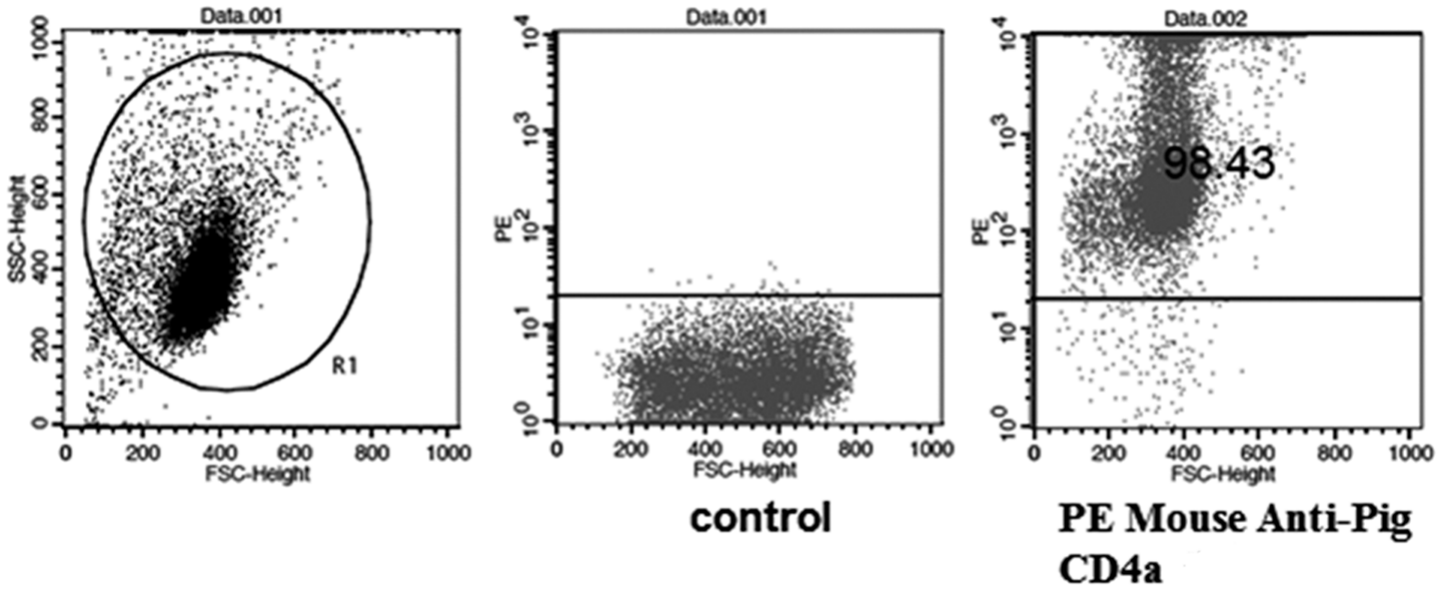
Purity of CD4^+^ T cells analyzed by flow cytometry. Porcine peripheral blood CD4^+^T cells were purified from PBMCs by immunomagnetic labeling of cells using mouse anti-pig CD4a monoclonal antibody and anti-mouse IgG MicroBeads. Cells were stained with PE Mouse Anti-Pig CD4a. Flow cytometry was performed using a FACSCalibur to analyze the purity of CD4^+^T cells.

CD4^+^ T cells were purified from PBMCs by magnetic activated cell sorting to obtain CD4^+^ T cells with greater than 98% purity (Fig 7).

**Fig 8:**
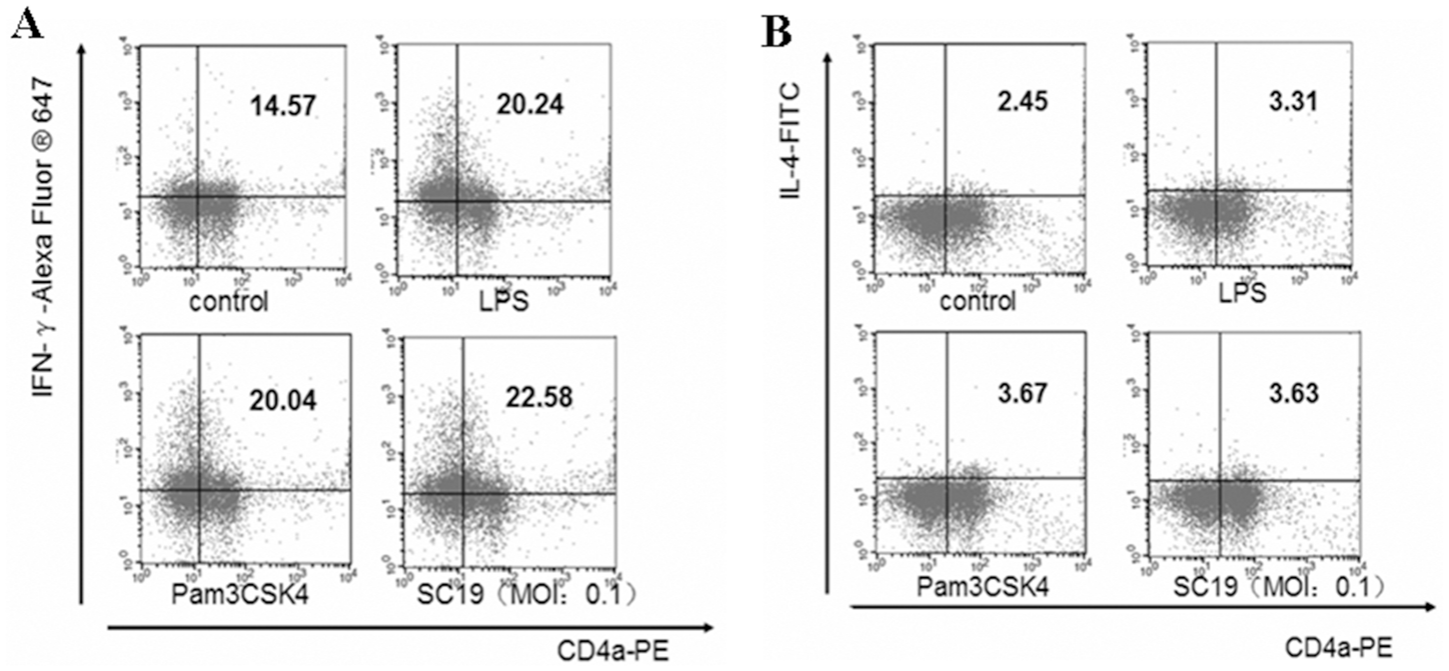
Differentiation of CD4^+^ T cells co-cultured with MoDCs and SC19. (A) The proportion of Th-1 cells. (B) The proportion of Th-2 cells. CD4^+^T cells were mixed with 5-day old MoDCs in the proportion of 4:1, both of them were stimulated by SC19 (MOI = 0.1); after 12 h, cells were analyzed by flow cytometry.

When MoDCs mixed with CD4^+^ T cells were stimulated by SC19 (MOI = 0.1) for 12 h, the proportion of Th-1 (CD4^+^, IFN-γ^+^) cells increased by 55% (Fig 8A).Further, the proportion of Th-2 (CD4^+^, IL-4^+^) cells increased by 48.2% (Fig 8B). However, the absolute level of Th-2 cells was much lower than that of Th-1. The results indicated that the SC19-stimulated MoDCs could mainly induce Th-1 differentiation.

### CD4+ T cells cytokine response to SC19-stimulated MoDCs

**Fig 9:**
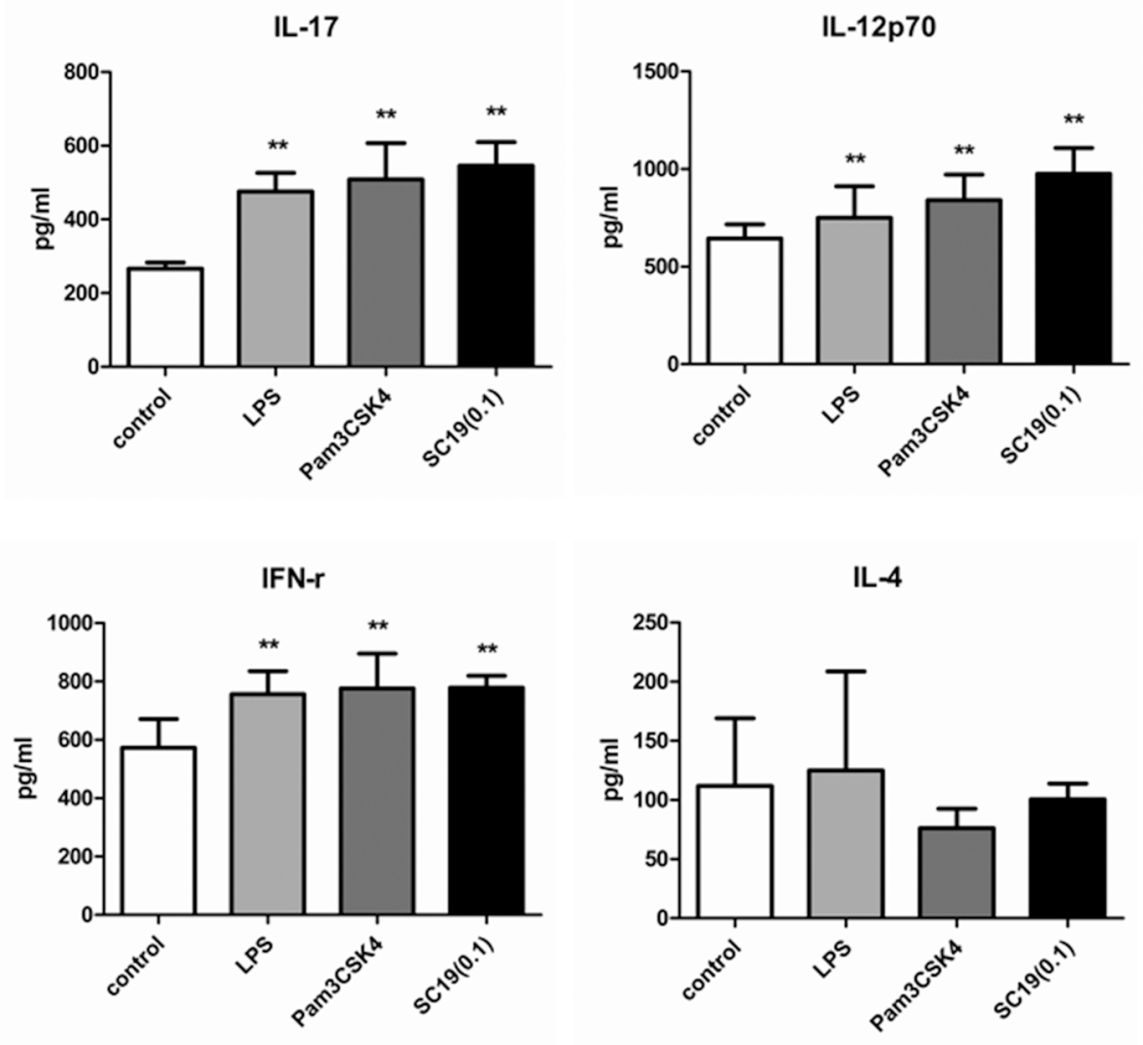
Cytokine in the supernatants of CD4^+^ T cells and MoDCs stimulated by SC19. CD4^+^ T cells were mixed with 5-day old MoDCs in the proportion of 4:1. The mixed cells were stimulated by SC19 (MOI = 0.1), LPS (1 μg/mL) or Pam3CSK4 (500 ng/mL) at 37°C with 5% CO_2_. 17 hours later, supernatant from each well were collected and analyzed for IL-4, IL-17, IL-12p70, and IFN-γ using Human Th1/Th2/Th17 Antibody Assay.

A mixture of MoDCs and CD4^+^ T cells was stimulated by SC19 (MOI = 0.1) for 17 h, and the levels of IL-4, IL-12p70, IL-17, and IFN-γ in the supernatants are shown in Fig 9. As expected, IL-12p70 was up-regulated by 51%, IFN-γ by 36% (based on the high basal level of 575 pg/mL), and IL-17 by more than 100%.

Compared with the results shown in Fig 6, it can be deduced that IFN-γ was mainly secreted by CD4^+^ T cells (Th-1 cells), and IL-12p70 was produced mainly by the MoDCs. The evident increase of IL-17 indicated that MoDCs might also induce Th-17 cells differentiation. IL-4 levels did not change noticeably. Thus, the results indicated that the SC19-stimulated MoDCs mainly induce Th-1 polarization.

## Discussion

*S*.*suis* is increasingly being recognized as an important pathogen for swine and human health. Serotype 2 (SS2) is the most common and most virulent among the 33 serotypes of *S*.*suis* [16]. *S*.*suis* infection in humans typically produces a purulent or nonpurulent meningitis, endocarditis, cellulitis, peritonitis, rhabdomyolysis, arthritis, spondylodiscitis, pneumonia, uveitis, endophthalmitis, and occasionally septic shock [17, 18, 19]. SS2 infection can cause mortality even in the Intensive Care Unit (ICU), with exaggerated level of Th-1 immune response [10, 20]. It is well known that MoDCs can stimulate Th-1 cell differentiation, but its role in induction of high level of Th-1 immune responseduring SS2 infection remains unknown [21]. In this study, CD14^+^ monocytes were purified from porcine PBMCs, and further directionally cultured to obtain MoDCs [15, 22]. SC19 strain of *S*. *Suis* serotype 2 was selected to stimulate MoDCs.

Firstly, the purity of porcine PBMC derived MoDCs was ascertained by their morphology, surface markers, and phagocytic and T cell proliferation promotion ability.

As expected, the phagocytic ability of the MoDCs increased to the peak at day 6 of culture and then decreased, and the surface makers SLA-II and CD172 were found to be 86.63% and 92.81% respectively at day 5 of culture. However, CD1 expression ranged between 20.14% and 33.90% at day 5 of culture. This was much lower than expected, given the purity of the MoDCs, and was inconsistent with suggestion that CD1 expression represented the purity of porcine MoDCs [15, 23]. Hence, this study also indicateed that CD1 expression may vary according to the origin of the DCs.

Secondly, SC19 was used to stimulate 5-day old porcine MoDCs and the response was measured. It was found that SC19 had no effect on the expression of CD172 and SLA-II on MoDCs; this may due to the high basal level of CD172 and SLA-II of porcine MoDCs. SC19 stimulation could down-regulated the phagocytic ability of MoDCs and then promoted T cell proliferation through MoDCs. Hence, SC19 infection could promote the maturation of porcine MoDCs, and the matured MoDCs could then promote T cell immune response. We also measured pro-inflammatory cytokines (IL-1β, IFN-γ, TNF-α, and IL-8), anti-inflammatory cytokines (IL-10, TGF-β1, IL-4), and IL-12 in the supernatants of the SC19-stimulated MoDCs. We found that the SC19-stimulated MoDCs produced high levels of pro-inflammatory cytokines and Th-1 type cytokine (IL-12) at 12 and 24 h, implying that the SC19 treated MoDCs could promote Th-1 polarization and inflammatory response.

Finally, CD4^+^ T cells were co-cultured with the SC19-stimulated MoDCs, and Th-1 polarization was evaluated. We found that the Th-1 (CD4^+^, IFN-γ^+^) cells increased at 12 h, and the levels of IFN-γ, IL-17 and IL-12p70 increased markedly. This demonstrated that the SC19-stimulated MoDCs can indeed induce Th-1 differentiation. Therefore, the present study indicated that SS2 infection could induce the maturation of MoDCs, which further induce CD4^+^ T cell differentiation towards Th-1 cells.

Since, STSLS patients showed an exaggerated level of Th-1 immune response, and the SC19-stimulated MoDCs can also induce such a high level of Th-1-immune response, it led us to consider that MoDCs may play an important role in the severe inflammation caused by SC19 infection. Based on the data from this study, it is reasonable to believe that MoDCs contribute to STSLS in the following three respects:

1. The SC19-stimulated MoDCs produce high levels of inflammatory cytokines (IL-1β, IL-8, TNF-α, and IFN-γ). DC derived IL-8 and IFN-γ recruit neutrophils and macrophages to migrate to the sites of inflammation, and these cells secrete large amounts of pro-inflammatory cytokines, resulting in increased inflammation [24]. IL-1β and TNF-α secreted by DCs induce vasodilation, and inflammatory cells migrate to the site of inflammation faster [25].
2. The SC19-stimulated MoDCs induce Th-1 cell differentiation, and Th-2 cell differentiation is suppressed by Th-1 cells derived IFN-γ. The suppressed Th-2 immune response, therefore, cannot stop the inflammation mediated by Th-1 cells [14, 26], while macrophages activated by IFN-γ promote inflammation [27].
3. The SC19-stimulated MoDCs produce a small amount of IL-10 and TGF-β1which suppress inflammation. The lack of IL-10 and TGF-β1 resulted in uncontrolled inflammation [28].

The SC19-stimulated MoDCs exaggerate inflammatory response and cause severe inflammation, which can further result in serious damage of tissues and organs, and finally the death of the host [29]. The interaction of physical and chemical factors is much more complex *in vivo*, and the real contribution of MoDCs to the inflammation storm and its relationship with other immune cells needs further experiments to be understood better.

## Materials and Methods

### Bacterial strain and growth conditions

SC19 is a highly virulent strain of SS2, originally isolated from a pig infected by *S*. *suis* in Ziyang, Sichuan [30]. SC19 was grown on Tryptic Soy Agar (TSA) plates at 37°C. Isolated colonies were used as inocula for Todd-Hewitt Broth (THB) with 10% fetal bovine serum (FBS), and were incubated for 8 h at 37°C with shaking. Working cultures were obtained by inoculating 100 μL of these cultures in 30 mL of THB and incubating for 16 h at 37°C with shaking. The number of CFU/mL in the final suspension was determined by spread plate method on Todd-Hewitt Agar (THA). Bacteria were washed twice in phosphate-buffered saline (1×PBS, pH 7.3) and were pre-opsonized using 20% fresh complete normal pig serum in 1×PBS (pH 7.3)[31]. Opsonization was performed for 30 min at 37°C with shaking. Bacteria were washed twice and appropriately diluted in complete cell culture medium.

### Animals

Porcine peripheral blood was obtained from 6–8 weeks old, healthy large white pigs raised at the Jingpin Farm (Huazhong Agricultural University, Hubei, China). None of the pigs was sacrificed. In order to ameliorate the suffering of the pigs, this experiment was performed in strict accordance with the Guide for the Care and Use of Laboratory Animals Monitoring Committee of Hubei Province, China, and the protocol was approved by the Committee on the Ethics of Animal Experiments at the College of Veterinary Medicine, Huazhong Agricultural University. The blood and sera derived from pigs were free of *S*.*suis* and other pig pathogens.

### Isolation of CD14^+^ monocytes and generation of MoDCs

Peripheral blood samples were collected into 50 mL centrifuge tubes with 0.5% heparin sodium, diluted in a ratio of 1:2 with sterile 1×PBS, overlaid on Ficoll-Hypaque (TBD science, Tianjin, China) and PBMCs were collected as previously detailed [32]. CD14^+^ monocytes were purified from PBMCs by immunomagnetic labeling of cells using mouse anti-pig CD14 monoclonal antibody (AbD Serotec, Raleigh, NC, USA) and anti-mouse IgG MicroBeads (Miltenyi Biotec, Bergisch-Gladbach, Germany) according to the manufacturer’s protocol. The CD14^+^ monocytes were cultured for 5 days in RPMI-1640 medium supplemented with 10% fetal bovine serum (FBS), 25 ng/mL recombinant porcine IL-4 and 10 ng/ml recombinant porcine GM-CSF (R&D systems, Minneapolis, MN, USA). The cell culture medium was replenished every 3 days. The typical veiled morphology of the cells was checked for every day. After five days of culture, CD1, SLA-II, and CD172a markers on these cells were analyzed by flow cytometry using monoclonal antibodies [33, 34].

### Flow cytometric analysis of MoDCs

After the CD14^+^ monocytes were cultured for 5 days (5-day old MoDCs) as mentioned above, 10^6^ cells were harvested for cytometric analysis.

6-day old MoDC were treated with LPS (1 μg/mL, Sigma Chemical, Poole, Dorset, UK), Pam3CSK4 (500 ng/mL, InvivoGen, San Diego, CA, USA), and SC19 (MOI = 0.1) for 24 h each and then 10^6^ cells from each treatment, along with control cells, were harvested for cytometric analysis.

The cells mentioned above were stained with mouse anti-pig CD1 (Southern Biotech, Cambridge, UK), mouse anti-pig SLA-II (MHC-II, AbD Serotec), mouse anti-porcine CD172a (SWC3, Antigenix America, Huntington Station, NY, USA) and analyzed on a FACSCalibur (BD Biosciences, Hiedelberg, Germany) by using CellQuest Pro software (BD Biosciences).

### MoDCs phagocytosis assay

MoDCs (2 × 10^6^ cells/mL) of different ages (2 d, 4 d, 6 d, and 8 d) were seeded into 24-well plates (0.5 mL per well); 0.5 mL of 0.1% neutral red stroke-physiological saline solution was added into each well and the cells were cultured at 37°C with 5% CO_2_. After 2 h, the cells were washed twice with 1× PBS, following which 0.5 mL/well 1% SDS was added to dissolve the cells. After 2 h at room temperature, OD570nm of the dissolved cells from different wells was detected by using a microplate reader (Bio Teke Corporation, Beijing, China).

Six-day old MoDCs were selected to be infected with pre-opsonized SC19 (MOI = 0.1) and two days later, OD570nm of the dissolved cells was detected by using a microplate reader (Bio Teke).

### Proliferation of T cells assay

Five-day old MoDCs were seeded into 24-well plates at 10^6^ cells/mL/well. MoDCs were incubated with SC19 (MOI = 0.001), LPS(1 μg/mL, Sigma) or Pam3CSK4(500 ng/mL, InvivoGen) for 24 h each at 37°C with 5% CO_2_. Mitomycin C was added into each well at final concentration of 50 μg/mL. MoDCs were washed twice in 1× PBS after 45 min for the subsequent steps.

PBMCs were isolated and seeded in cell culture dishes for 2 h and the suspended cells were collected as T cells. T cells were seeded in 96-well plates at 3×10^5^ cells/mL, 100 μL per well.

The treated MoDCs were added into 96-well plates with T cells in different proportions (1:5, 1:10, 1:100). T cells alone, MoDCs alone and medium alone wells were set as controls. Three days later, 10 μL CCK-8 (Dojindo Laboratories, Japan) was added in each well and further incubated for 5 hours at 37°C with 5% CO_2_. OD450nm of each well was detected by using a microplate reader. SI (Stimulation Index) was calculated as following:

$$SI=\frac{(DC+T)OD450nm-(DC)OD450nm}{(T)OD450nm-(medium)OD450nm}$$

### MoDCs cytokine assay

5-day old MoDCs were stimulated by SC19 (MOI: 0.1, 0.01, 0.001), LPS (1 μg/mL) or Pam3CSK4 (500 ng/mL), at 37°C with 5% CO_2_. Supernatants were collected after 12 and 24 h of stimulation and analyzed for cytokines (IL-1β, IL-4, IL-6, IL-10, IL-12, TNF-a, IFN-γ, TGF-β, and GM-CSF) using Porcine Cytokine Array according to the manufacturer’s protocol (RayBiotech, Norcross, GA, USA).

### Isolation of porcine peripheral blood CD4+T cells

Porcine peripheral blood CD4^+^T cells were purified from PBMCs by immunomagnetic labeling of cells using mouse anti-pig CD4a monoclonal antibody (AbD Serotec) and anti-mouse IgG MicroBeads (Miltenyi Biotec). Cells were stained with PE mouse anti-pig CD4a (BD Biosciences); after washing, cells were re-suspended in sorting buffer for FACS analysis. Flow cytometry was performed using a FACSCalibur (BD Biosciences), and the purity of CD4^+^ T cells was demonstrated to be 98%.

### Differentiation and cytokine assay

CD4^+^T cells were mixed with 5-day old MoDCs in the proportion of 4:1. The mixed cells were seeded into 24-well plates at 10^6^ cells/mL/well, and stimulated by SC19 (MOI = 0.1), LPS (1 μg/mL) or Pam3CSK4 (500 ng/mL) at 37°C with 5% CO_2_.

After stimulation for 12 h, 4 μL of PMA/Ionomycin/BFA/Monensin mixture was added to each well. After 5 hours, supernatant from each well was collected and analyzed for cytokines (IL-4, IL-17, IL-12p70, and IFN-γ) using Human Th1/Th2/Th17 Antibody Array, according to the manufacturer’s protocol (RayBiotech). The collected cells were stained with the following monoclonal antibodies: PE mouse anti-pig CD4a (BD Biosciences), Alexa Fluor^®^647 mouse anti-pig IFN-γ (BD Biosciences) and mouse anti-bovine interleukin-4: FITC (AbD Serotec).

### Statistical analysis

The data were expressed as mean±standard deviations. Data were analyzed using Student’s *t*-test. P values were derived to assess statistical significance and are indicated in the figure panels. The significance level for all analyses was set to P< 0.05.
